# Macrogenomic Analysis Reveals Soil Microbial Diversity in Different Regions of the Antarctic Peninsula

**DOI:** 10.3390/microorganisms12122444

**Published:** 2024-11-27

**Authors:** Jiangyong Qu, Xiaofei Lu, Tianyi Liu, Ying Qu, Zhikai Xing, Shuang Wang, Siluo Jing, Li Zheng, Lijun Wang, Xumin Wang

**Affiliations:** 1 College of Life Science, Yantai University, Yantai 264005, China; qjy@ytu.edu.cn (J.Q.); luxiaofei18@outlook.com (X.L.); liutianyi0719@163.com (T.L.); quuuying@163.com (Y.Q.); xingzhk@ytu.edu.cn (Z.X.); wangshuang0456@126.com (S.W.); jingsiluo@icloud.com (S.J.); 2 First Institute of Oceanography, Ministry of Natural Resources, Qingdao 266061, China; zhengli@fio.org.cn

**Keywords:** South Shetland Island, soil microorganisms, macrogenome, community structure

## Abstract

(1) Background: The unique geographical and climatic conditions of the Antarctic Peninsula contribute to distinct regional ecosystems. Microorganisms are crucial for sustaining the local ecological equilibrium. However, the variability in soil microbial community diversity across different regions of the Antarctic Peninsula remains underexplored. (2) Methods: We utilized metagenome sequencing to investigate the composition and functionality of soil microbial communities in four locations: Devil Island, King George Island, Marambio Station, and Seymour Island. (3) Results: In the KGI region, we observed increased abundance of bacteria linked to plant growth promotion and the degradation of pollutants, including PAHs. Conversely, Marambio Station exhibited a significant reduction in bacterial abundance associated with iron and sulfur oxidation/reduction. Notably, we identified 94 antibiotic resistance genes (ARGs) across 15 classes of antibiotics in Antarctic soils, with those related to aminoglycosides, β-lactamase, ribosomal RNA methyltransferase, antibiotic efflux, gene regulatory resistance, and ABC transporters showing a marked influence from anthropogenic activities. (4) Conclusions: This study carries substantial implications for the sustainable use, advancement, and conservation of microbial resources in Antarctic soils.

## 1. Introduction

As the fifth largest continent in the world, Antarctica has unique climatic characteristics and its polar environment plays a crucial role in the global climate balance. The Antarctic Peninsula, located in West Antarctica, is the largest peninsula on the continent and the furthest reach into the ocean, reaching a latitude of 63° S. It is warmer and receives higher precipitation than the rest of the Antarctic, and its complex geology makes the Antarctic Peninsula a key location for research on Antarctic ecosystems, biogeography, and the effects of climate change. The Marambio Station, established in 1969, has carried out long-term environmental monitoring work here. The high level of human activity here provides an ideal environment for studying the effects of human disturbance on microbial communities [1]. Seymour Island, located at the northern tip of the Antarctic Peninsula, is known for its rich paleontological finds, especially the fossil record from the Paleocene to Eocene periods, which have provided scientists with important clues to understanding past environmental changes on the Antarctic Peninsula [2]. King George Island sits atop a volcanic arc that experienced activity throughout the Mesozoic and most of the Cenozoic eras, located on the western edge of the Antarctic Peninsula. The region features multiple dyke systems, primarily belonging to subalkaline series, with calc-alkaline and transitional characteristics. While King George Island lacks active volcanoes, it showcases various dormant volcanic landforms, including dykes and sequences of volcanic rocks. These geological attributes create an exceptional natural laboratory for investigating microbial diversity. Notably, the interplay of volcanic and glacial settings offers a distinctive avenue to explore microorganisms thriving in extreme conditions [3,4,5,6]. Devil’s Island derives its name from the two prominent peaks flanking the valley, characterized by its low-lying and angular topography. The landscape primarily features loose gravel, alongside a notably elevated beach on the northern shore, which showcases a unique volcanic rock face. Recently, the surge in Antarctic tourism has heightened the effects of human activities on this site [7]. Research at these sites will enhance our understanding of Antarctic Peninsula ecosystems.

Research on the Antarctic Peninsula in recent years has focused on climate change and ecosystem change, but also on the impact of human activities on the region. Moreover, pollution events such as oil spills, invasive alien species, and eutrophication not only indirectly change ecological conditions, but also have a direct impact on the structure and function of microbial communities [8,9,10,11]. It has been shown that there is a significant correlation between certain microbial populations and ice-cover conditions (e.g., seaweeds are positively correlated with ice cover, whereas *Spirulina* is negatively correlated) and temperature changes (e.g., *Cryptococcus* is positively correlated with temperature increase, whereas *Spirulina* is negatively correlated). In recent years, polar snow algae have shown a tendency to proliferate rapidly, and the phenomenon of pink “watermelon snow” has even appeared in the Antarctic region. These snow algae are adapted to cold environments and are usually dormant at low temperatures, but will proliferate rapidly when temperatures rise. The snow takes on a reddish color because the algae contain carotenoids that help protect them from ultraviolet light. This phenomenon suggests that warmer temperatures in the Antarctic are already having a clear visible effect [12,13]. These findings emphasize the high sensitivity of microbial communities in the face of ecological change and highlight the importance of studying them in depth [14]. These phenomena highlight the high sensitivity of microbial communities in response to ecological changes and the importance of their intensive study. However, current research is still limited, especially the lack of comprehensive comparative studies of different regions of the Antarctic Peninsula.

This study addresses knowledge gaps regarding soil microbial diversity across four unique regions of the Antarctic Peninsula. Previous research depended on 16S rRNA gene library techniques and denaturing gradient gel electrophoresis (DGGE) to identify soil microorganisms. However, these methods often reveal a narrow spectrum of dominant microbial taxa. Recent advancements in DNA sequencing, particularly macrogenome sequencing, provide a broader perspective on soil microbial composition and their functional characteristics. This technology enables the discovery of a wider variety of microbial species and clarifies their roles in the ecosystem. Thus, it significantly improves our understanding of the structure and function of microbial communities [15,16]. We collected 26 soil samples from Marambio Station, Seymour Island, King George Island, and Devil Island during the 31st Chinese Antarctic Scientific Expedition. Through bioinformatics analysis of macrogenomic data from multiple samples obtained by high-throughput sequencing technology, this study assessed the species diversity and functional characteristics of bacterial communities in soil. This study paid particular attention to the microbial community profiles of different geographic locations and their comparisons with each other, and explored the differences between various types of regions.

## 2. Materials and Methods

### 2.1. Sites and Sampling

**Figure 1:**
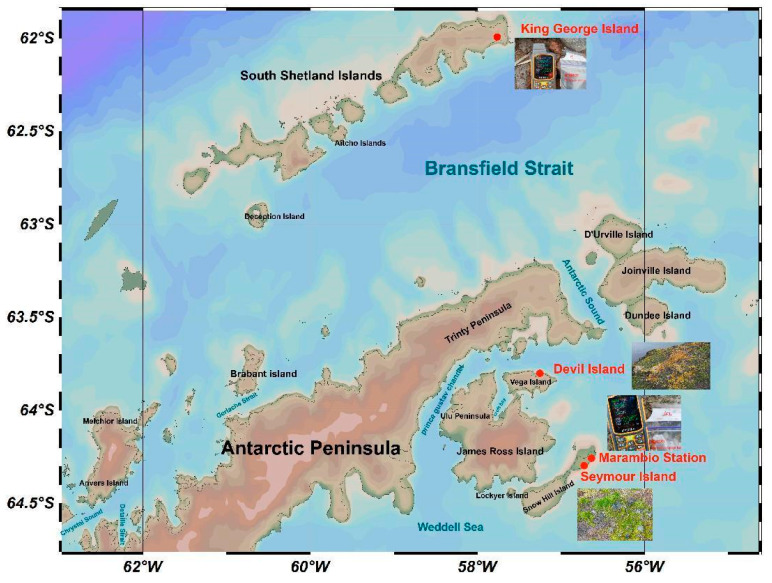
Map of Antarctic Peninsula and South Shetland Island indicating sampling sites. The red dots in the figure are abbreviated to represent sampling areas.

Samples were taken from four areas of the Antarctic Peninsula: Devil Island (DI), King George Island (KGI), Marambio Station (MS), and Seymour Island (SI) (Figure 1). Differences in sample sizes between sites were mainly due to the fact that the geographic and climatic conditions of the different sites affected sample collection to varying degrees. Twelve soil samples were collected from the KGI area, four from SI, eight from MS, and two from DI. The sampling process involved the use of a sterile shovel to collect surface soil samples 5–10 cm from the ground, and at each sampling point the samples were mixed from three replicates [17]. The mixed soil was packed and marked with sterile sealing bags, transported to the School of Life Sciences, Yantai University, under frozen (−20 °C) conditions, and stored in a −80 °C refrigerator before the experiment (see Table 1 or detailed sample information). Ocean Data View (ODV) software (v.5.7.2) was used to visualize the latitude and longitude position of each sampling area [18].

### 2.2. DNA Extraction and Sequencing

Total microbial DNA extraction from soil samples utilized the Fast DNA TM Spin Kit (MP Biomedicals, Guangzhou, China) per the manufacturer’s guidelines. Following this, the extracted DNA underwent rigorous quality assessment. We employed the Qiagen DNeasy Plant Mini Kit (Qiagen, Hilden, Germany) for additional extraction, adhering strictly to the provided protocols. The concentration of the isolated DNA varied between 50 and 100 ng/µL, as determined by the Qubit 3.0 Fluorometer (Thermo Fisher Scientific, Waltham, MA, USA). Throughout the extraction procedure, we maintained strict contamination control protocols. Specifically, we implemented negative controls, which consisted of blank reaction tubes containing only extraction reagents, and blank controls, devoid of any microbial samples, containing solely extraction buffer. These control samples underwent the same extraction procedure as the actual samples to monitor potential contamination throughout the process. Metagenomic shotgun sequencing libraries were constructed and sequenced at Shanghai Biozeron Biological Technology Co. Ltd. (Shanghai, China). In brief, for each sample, 1 μg of genomic DNA was sheared by a Covaris S220 Focused-ultrasonicator (Woburn, MA, USA) and sequencing libraries were prepared with a fragment length of approximately 450 bp. All sequencing was carried out on the Illumina Novaseq 6000 platform (Illumina, San Diego, CA, USA) in pair-end 150 bp (PE150) mode, with sequencing depths typically ranging from 5 GB to 10 GB per sample.

### 2.3. Data Quality Control

The raw sequence reads underwent quality trimming with Trimmomatic (v 0.36) [19] software to remove low-quality reads and adaptor contaminants, resulting in clean sequence reads. Single-sample assembly was then performed using Megahit (v1.2.8) [20] software, which merges short sequences from gene sequencing into longer contiguous sequences with a minimum contig length of 500. Megahit excels at assembling complex genomic data, providing improved integrity and continuity while requiring fewer resources, less time, and producing superior assembly results. Gene prediction was conducted on the assembled DNA sequences using Prokka (v1.13) [21] software. Predicted genes were then clustered and made non-redundant using CD-HIT (v4.8.1) [22] with the coverage threshold set at 90% and the similarity threshold set at 95% to construct a non-redundant gene set in order to create a unique gene set for further analysis. The nucleic acid sequences were translated into protein sequences using Transeq (v.6.6.0.0) [23] and quantified with Salmon (v.1.9.0) [24].

### 2.4. Species and Functional Annotation

Species annotation was done on clean reads using kraken2 (v.2.1.0) [25] software for obtaining annotated species information at various taxonomic levels like kingdoms, phyla, orders, families, genera, and species. Kraken2 is faster, more flexible in database selection, and more readable in results. Bracken (v.2.0) [26] software was then used for species taxonomic statistics and to build species abundance tables at each taxonomic level. Alpha diversity boxplots along with the top 10 phyla, 20 genera, and 20 species of each group were compared for differences. The non-redundant gene sets were compared with the KEGG, EggNOG, and CARD databases using the Diamond software (v.0.9.22.123) [27]. Functional annotation was carried out with Emapper (v.2.0.1) based on the comparison results. Abundance of this functional category was calculated by summing gene abundances for KO, COG, CAZY, and Resfam.

### 2.5. Statistical Analysis and Data Visualisation

The Shannon [28] and ACE [29] indices for alpha diversity were computed using the ‘vegan’ package (v.2.6-8) in R software (v.4.1.0). Alpha diversity assessment used the Kruskal–Wallis test with FDR correction (control level of 0.05) to determine differences between sampling sites. Non-metric multidimensional scaling (NMDS) analysis was conducted based on the Bray–Curtis distance algorithm, followed by the PERMANOVA test to assess community composition variability among grouped samples (https://www.bioincloud.tech/task-meta) (accessed on 12 November 2024). A two-sample t-test compared different groups, preceded by an f-test to evaluate group variances. Linear Discriminate Analysis Effect Size (LEfSe) was employed at the genus level of bacterial community abundance, selecting genera with an LDAScore exceeding 4 (http://huttenhower.sph.harvard.edu/galaxy/) (accessed on 12 November 2024). Pearson’s correlation coefficient analyzed relationships between microbiological and sampling area through SPSS (version 21.0). Data visualization and statistical analyses were carried out using Image GP (http://www.ehbio.com/ImageGP/) (accessed on 12 November 2024), Bioinformatics (http://www.bioinformatics.com.cn/) (accessed on 12 November 2024), and the R Software (v.4.1.3; R Core Team 2023) with the following packages: ggplot2 (v.3.5.0), tidyr (v.1.3.0), corrplot (v.0.92) magrittr (v.2.0.3), alongside various packages including tidyverse (v.2.0.0), psych (v.2.4.3), reshape (v.1.4.4), ggtree (v.3.12.0), aplot (v.0.2.2), and heatmap.

## 3. Results

### 3.1. Species Diversity Analysis

**Figure 2:**
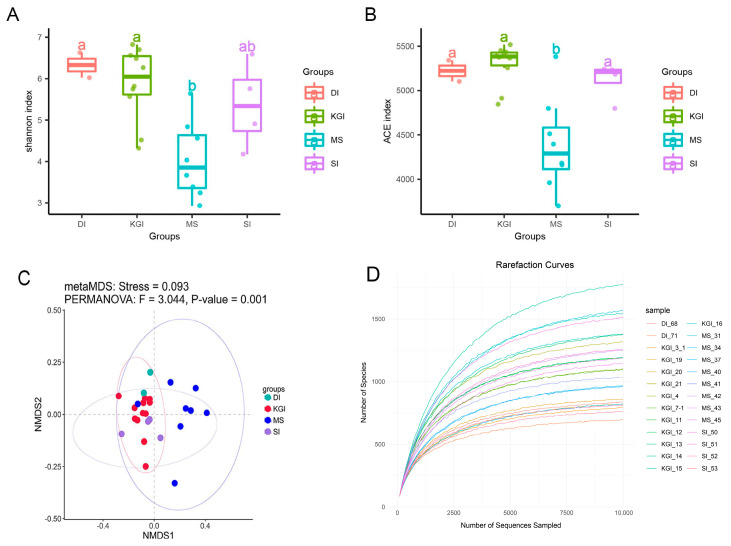
Statistical comparison of Shannon index (**A**) and ACE index (**B**) among soil microbial communities in four regions. The symbols “a” and “b” in the figure denote levels of statistical significance, indicating meaningful differences between groups. NMDS analysis of soil microbial communities in four regions (**C**). MetaMDS analysis generated a map (stress value of 0.093) effectively showing microbial community structure; a larger distance between sample points indicates greater difference in community composition, while a smaller distance indicates more similarity, PERMANOVA (F = 3.044, *p* < 0.001). (**D**) Rarefaction curves were estimated for sequencing data species for each sample.

In terms of sample coverage, species richness in the MS area remains significantly lower than in other areas of the Antarctic Peninsula. This includes a comparison with the closer SI, which exhibits a higher species richness and similar diversity to other regions, both nearby and distant. Regarding species evenness, MS also displayed similarly low levels. Structural differences between communities can be effectively visualized through non-metric multidimensional scaling analysis (NMDS), where distances on the graph reflect the level of beta diversity between samples. It is important to note that while the microbial communities around MS exhibited higher species diversity within the community, the microbial community structure of the Antarctic Peninsula as a whole remained more uniform. The species richness trend was evaluated using Rarefaction Curves, revealing that dilution curves flattened as sampling sequences neared 10,000. This indicates that additional sequences yield few new species, suggesting potential sequencing saturation (Figure 2).

### 3.2. Taxonomic Profiling of Soil Metagenomes of Antarctic Peninsula

**Figure 3:**
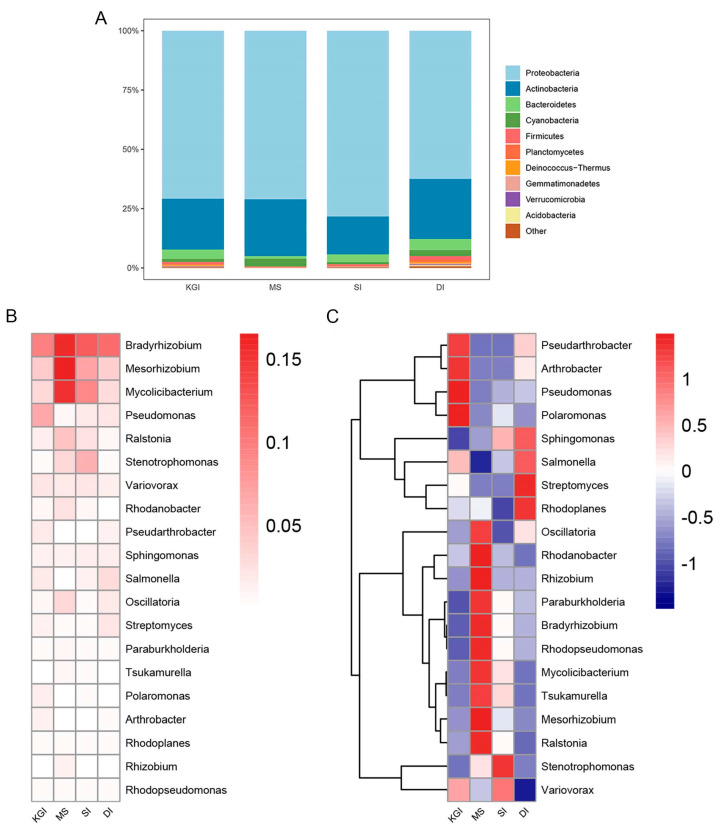
Relative abundance by percentile contribution of sequences identified on a phylum-rank taxonomic level (**A**). Relative abundance heatmap of dominant genera (top 20 in relative abundance) in bacterial communities. (**B**) Relative abundance according to sequence percentage value; color scale—relative abundance (%), (**C**) Scaling within genus rows across all examined samples; relative abundance above average—dark red, relative abundance below average—dark blue.

A total of 40 phylum-level taxonomic units were detected in soil samples from the four selected regions, with the four most abundant phyla being Ascomycetes, Actinobacteria, Anaplasma, and Cyanobacteria (Figure 3A). The relative abundance of Ascomycetes phylum in SI soils exceeded that of other areas. In contrast, the relative abundance of the phylum Actinobacteria in the soils of MS was lower than elsewhere, while the relative abundance of the phylum Cyanobacteria was higher. A total of 1335 genera-level mycobacterial genera were identified (Figure 3B,C). The dominant genera in the soil samples from MS included *Mesorhizobium* and *Mycolicibacterium*. The relative abundance of *Ralstonia*, *Rhodopseudomonas*, *Bradyrhizobium*, *Paraburkholderia*, *Rhizobium*, and *Rhodanobacter* genera in the soil samples from MS was higher than in other areas. On KGI, *Pseudarthrobacter*, *Arthrobacter*, *Pseudomonas*, and *Polaromonas* were the dominant genera. The main active genera in the soil samples from SI were *Variovorax* and *Stenotrophomonas*. It is worth noting that despite the high genus diversity in the DI area, it had the lowest number of dominant genera with only *Bradyrhizobium*, which was also notably present in all areas (Figure 3C).

**Figure 4:**
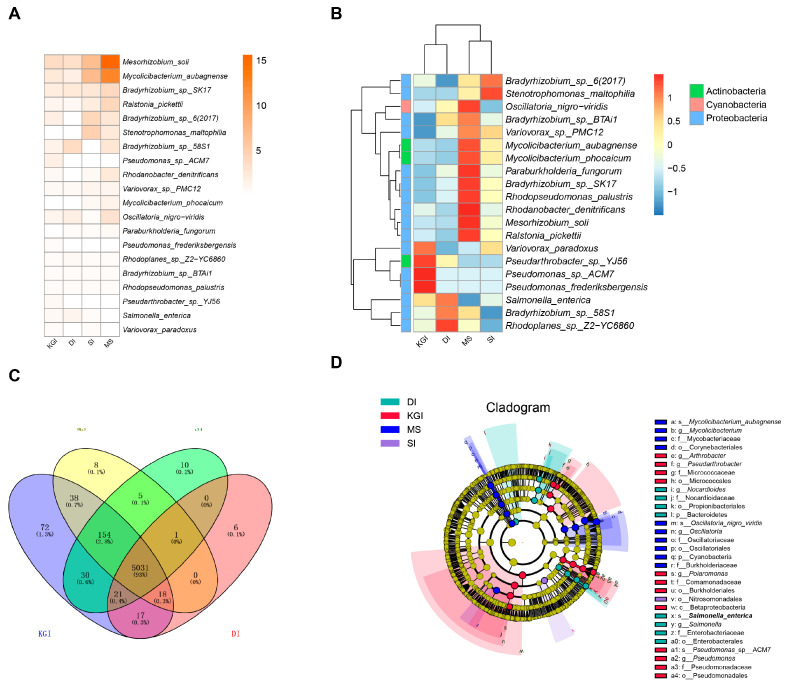
Relative abundance heatmap of sequences identified on a species-rank taxonomic level. (**A**) Relative abundance according to sequence percentage value; color scale—relative abundance (%). (**B**) Scaling within genus rows across all examined samples; relative abundance above average—red–orange, relative abundance below average—blue. (**C**) Venn diagram showing distribution patterns of different species in soil microbial communities belonging to four different sampling areas. (**D**) Cladogram displaying species with significant differences (LDA > 4) between groups.

**Figure 5:**
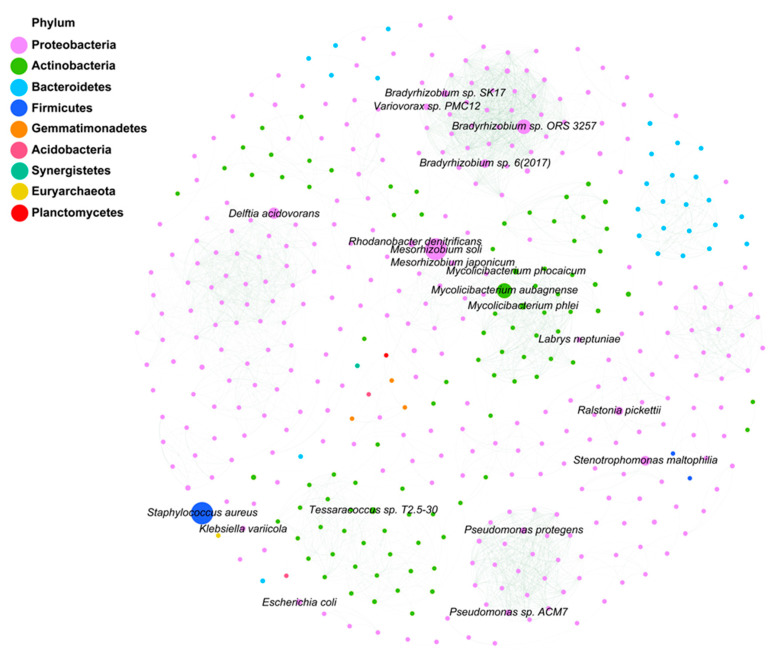
Microbial network analysis. Abundance top 500 species level abundance table for correlation analysis. Screening Pearson absolute value ≥ 0.9, *p*-value < 0.05 nodes for graphing. Node color represents species level species corresponding to different gate levels; green line indicates a positive correlation, red line indicates a negative correlation (results of negative correlation were not available). Node size indicates the abundance size of the corresponding species level. Only the top 20 species names were shown in the figure.

The total number of species detected in all samples amounted to 5678. The vast majority of the top 20 species in terms of relative abundance belonged to Proteobacteria. In addition to Proteobacteria, there were some notable members from other phyla: including Mycolicibacterium phocaicum from the genus *Cetobacterium*, *Mycolicibacterium* aubagnense from the genus *Cetobacterium* aubagnense, and *Pseudarthrobacter* sp. YJ56 from the genus *Pseudarthrobacter*, which all belong to the Actinobacteria phylum; while the Cyanobacteria phylum was represented by the black-green trematode *Oscillatoria nigroviridis* (Figure 4A,B). *Mesorhizobium soli*, *Mycolicibacterium* aubagnense, and *Bradyrhizobium* sp. SK17 were the dominant species (relative abundance > 1%) in each region. *Ralstonia pickettii* and *Bradyrhizobium* sp. 6 (2017) were dominant in KGI soil samples, SI soil samples, and MS soil samples. *Stenotrophomonas maltophilia* and *Variovorax* sp. PMC12 were the dominant species in soil samples from MS and SI. *Rhodanobacter denitrificans* and *Mycolicibacterium phocaicum* were the dominant species only in the soil samples from MS. The correlation analysis of the top 500 species in abundance was conducted (Figure 5). Nodes were screened based on Pearson absolute value ≥ 0.9 and *p*-value < 0.05 for mapping. There are 438 nodes in the graph with 3551 connectivity. Node colors indicate species levels corresponding to different phylum levels. Green lines show positive correlations. The high correlation between these species indicates a complex network of interactions, which includes symbiosis, competition, predation, and reciprocal symbiosis. These factors form a community structure dominated by Proteobacteria, Actinobacteria, Bacteroidetes, and Firmicutes.

Examining the number of shared and unique species across different regions in Venn diagrams reveals microbial community similarities and differences between samples or environments. The overall picture indicates generalized connectivity among the ecosystems of the Antarctic Peninsula, with as many as 5031 bacterial species shared across regions. Soil samples from the KGI area contained 72 unique endemic species, a high level of diversity compared to other areas. Six endemic species were identified in soils from the DI region, namely *Rickettsia asembonensis*, *Rickettsia heilongjiangensis*, *Candidatus Arthromitus* sp. SFB-mouse, *Planococcus* sp. ZOYM, *Geobacillus* sp. 1121, and *Nocardia* sp. C-14-1. On the other hand, ten endemic species were recorded in the soils of SI, including *Thermosipho* sp. 1070, *Borreliella finlandensis*, *Stenotrophus* sp. *Finlandensis*, *Stenotrophomonas nitritireducens*, *Pseudomonas* sp. THAF42, *Marinobacter* sp. THAF197a, *Rickettsia conorii*, *Staphylococcus* sp. CDC3, *Staphylococcus* species, *Streptomyces* sp. RLB3-5, *Streptomyces* sp. Y27, and *Corynebacterium* sp. L2-79-05. Eight endemic species were identified in the soils of MS, including *Thermotoga* sp. RQ2, *Citrobacter* sp. RQ2, Citrobacter sp. ABFQG, *Shewanella* sp. 33B, *Bacillus intestinalis*, *Bacillus* sp. SJ-10, *Microscilla* sp. PRE1, *Streptomyces flavovirens*, and *Streptomyces* sp. x4 (2010). The results of LEfSe analysis showed that 8, 12, 10, and 1 biomarkers were identified in the DI, KGI, MS, and SI groups, respectively (Figure 4D). In the extreme environment of Antarctica, only Mycolicibacterium showed a higher relative abundance in the MS area, while the other four genera showed the opposite trend.

### 3.3. Functional Composition and Pathways of Soil Microorganisms in the Antarctic Peninsula Region

**Figure 6:**
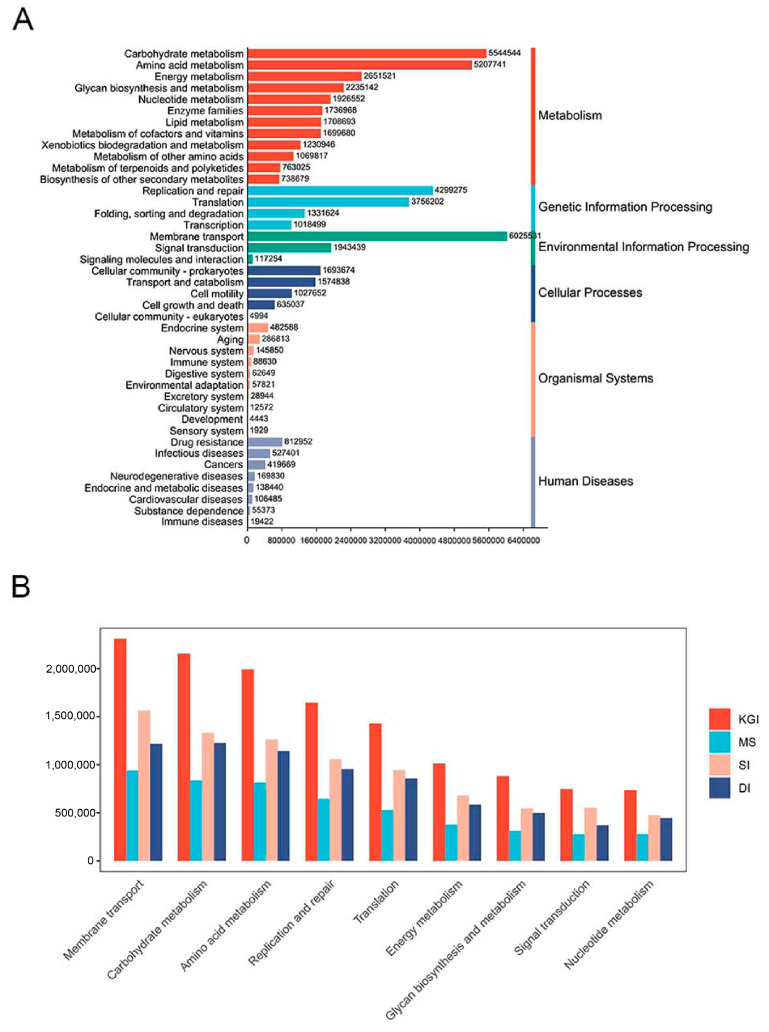
Statistical chart of KEGG pathway related functional genes (**A**). Distribution of the TOP 10 functional categories at KEGG level 2 in four different regions (**B**).

KEGG annotation showed that the pathways with a high number of genes involved were mainly in membrane transport (11.36%), carbohydrate metabolism (10.43%), amino acid metabolism (9.77%), replication and repair (8.03%), translation (6.95%), energy metabolism (4.93%), glycan biosynthesis and metabolism, signal transduction, nucleotide metabolism, etc. These functional classifications were all least abundant in soil samples from the MS area (Figure 6A,B). The annotated genes in each region were categorized and statistically. Among them, the DI area soil group had a total of 7133 genes, the KGI area soil group presented 10,377 genes, the MS area soil group presented 8035 genes, and the SI area soil group presented 8362 genes. There were 6662 core genes in the four groups, accounting for 62.3%. In addition, each group had unique genes, which were 1731 (16.2%), 28 (0.3%), 105 (1%), and 50 (0.5%), sorted by KGI area soil, DI area soil, SI area soil, and MS area soil, respectively. The highest number of unique genes was in the KGI region.

**Figure 7:**
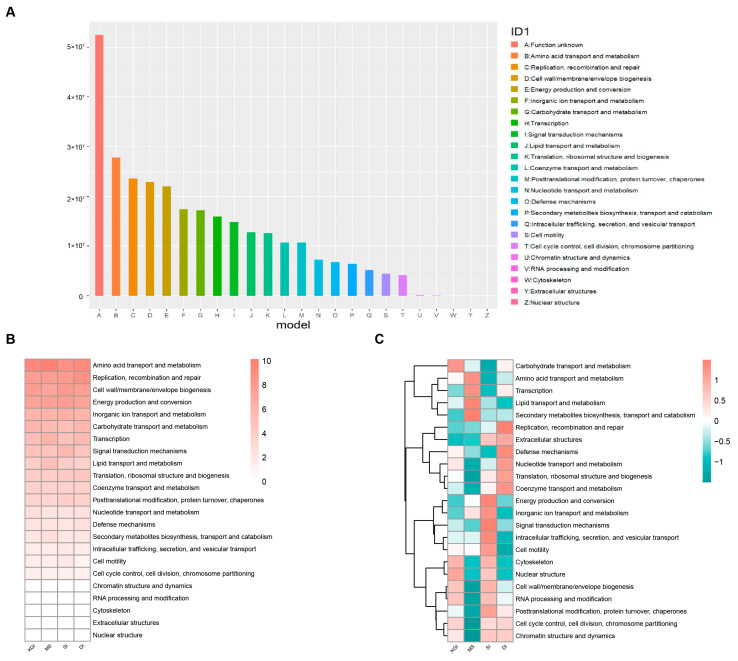
Functional classification of microorganisms in soils from four regions of the Antarctic Peninsula in the eggNOG functional database (**A**). Relative abundance of COG in soil microbial communities from four regions of the Antarctic Peninsula (**B**). Relative abundance according to sequence percentage value; color scale—relative abundance (%) (**C**). Scaling within rows across all examined samples.

The COG classified 24 functional descriptions in these four Antarctic soil samples. The four dominant functions that accounted for the majority of functions were B: amino acid transport and metabolism, C: replication, recombination, and repair, D: biosynthesis of cell wall/cell membrane/membrane structures, and E: energy production and conversion, with percentages of 9.40%, 7.97%, 7.74%, and 7.45%, respectively (Figure 7A). The relative abundance of amino acid transport and metabolic functions was highest in soil samples from MS. The relative abundance of replication, recombination, and repair functions was clearly higher in soil samples from the DI area than in soil samples from other areas. The relative abundance of cell wall/membrane/envelope biogenesis functions was higher in soil samples from KGI and SI and lowest in soil samples from MS. The relative abundance of energy production and conversion functions was highest in soil samples from SI (Figure 7B,C). Furthermore, it is worth noting that a significant (*p* < 0.05) difference was found between the soil samples from the MS and SI soil samples as a function of cell motility by rank-sum test results.

**Figure 8:**
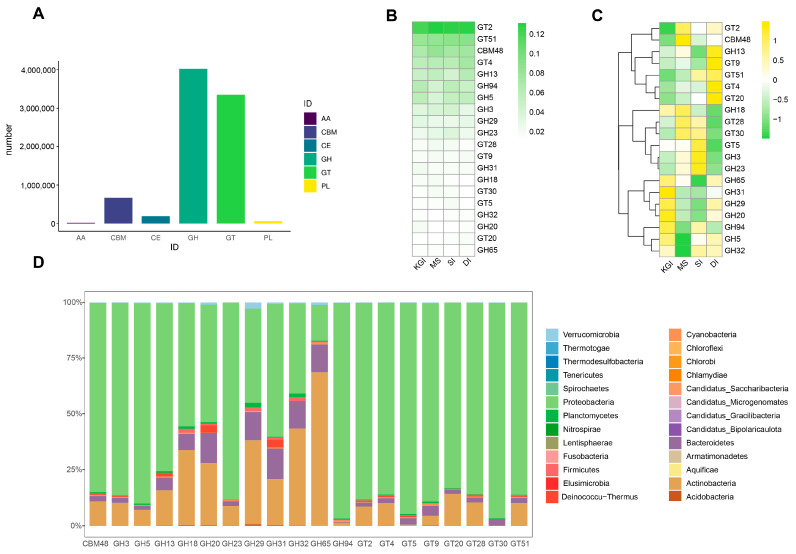
Carbohydrate-active enzyme analysis of soil microorganisms in four areas of the Antarctic Peninsula (**A**). Heatmap for the analysis of differences in soil microbial carbohydrate-active enzyme families (TOP20) in four different regions of the Antarctic Peninsula (**B**). Relative abundance according to sequence percentage value; color scale—relative abundance (%) (**C**). Scaling within rows across all examined samples. Microbial abundance corresponding to high abundance carbohydrate-active enzyme families (top 20) (**D**).

The CAZy database includes five categories and one related module. Its main components are: auxiliary oxidoreductases (AAs), carbohydrate-binding modules (CBMs), carbohydrate esterases (CEs), glycoside hydrolases (GHs), polysaccharide lyases (PLs), and glycosyltransferases (GTs) (Figure 8A). The percentages of AAs, GTs, CEs, GHs, PLs, and CBMs in soil samples from these four different regions of Antarctica were 0.24%, 40.23%, 2.29%, 48.41%, 0.74%, and 8.06%, respectively. Each category was divided into different families. In the present study, GHs that mainly hydrolyze and rearrange glycosidic bonds presented 58 families. There are 69 families of GTs, which are mainly involved in the formation of glycosidic bonds.

Heatmaps were drawn for the top 20 families in terms of relative abundance (Figure 8B,C). Of these, GT2 had the highest relative abundance in soil microbial samples from these four regions of Antarctica, particularly in the MS area. The relative abundance of GT51 and CBM48 was also high in each region, with the highest relative abundance of GT51 at DI and CBM48 at MS. Relative abundances of GH31, GH29, GH20, GH94, and GH5 are also high in the KGI area. The relative abundance of GT4, GT9, GT20, and GH13 was also high on DI. Relative abundances of GT28, GT30, GH3, and GH23 were also high in samples from SI and MS. Among the differential families of carbohydrate-active enzymes, the abundances of families GH29, GT35, GH32, GH95, and GH77 were generally higher in soil samples from the SI area than from the MS area, with the exception of a few sampling sites. The microorganisms corresponding to the top 20 families were further analyzed. The relative abundance of Actinobacteria in the GH65 family was significantly higher than in the other families. The relative abundance of Bacteroidetes was higher in family GH20, GH29, GH31, GH32, and GH65. The relative abundance of Proteobacteria in the GH94 and GT30 families was higher than in the other families (Figure 8D).

**Figure 9:**
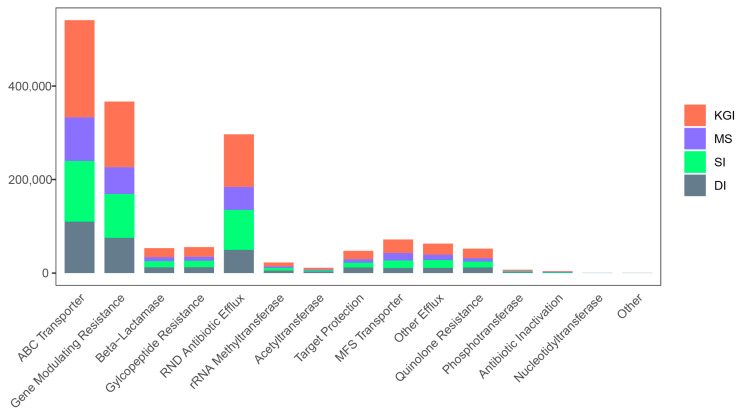
Analysis of soil antibiotic resistance gene resistance mechanisms in four different regions of the Antarctic Peninsula.

The Resfam database analysis results indicated that there were 15 classifications of mechanisms with 95 ARGs (Figure 9). Of these, ABC Transporter (33.95%), Gene Modulating Resistance (23.02%), and RND Antibiotic Efflux (18.63%) mechanisms were more abundant in soil samples from these four regions of Antarctica. ARGs (e.g., *macB*, *RNDAntibioticEffluxPump*, *ABCAntibioticEffluxPump*, *vanS*, *vanR*, *msbA*) were more abundant than others in Antarctic Peninsula soils. The KGI area had the highest number of ARGs in the soil samples with an average of 609,104 ARGs per soil sample. The DI, MS, and SI areas had an average of 315,209 ARGs, 269,082 ARGs, and 404,692 ARGs per soil sample, respectively. Soil samples from the MS area had the lowest number of resistance genes, mainly due to the lowest species richness and species diversity of the bacterial community in this area. Although species richness and species diversity of the bacterial community was higher in the DI area, the abundance of antibiotic resistance genes was lower in the soil samples.

Due to the long-term presence of human activities at MS, this study analyzed the abundance of antibiotic resistance genes in soil samples from two nearby locations: MS and SI. We rank-summed the samples. The results revealed significant differences in the abundance of ARGs between the two sample sets, as depicted in Table 2. These significantly different ARGs (*p* < 0.05) were strongly influenced by anthropogenic factors. We categorized their resistance mechanisms into acetyltransferases, β-lactamases, rRNA methyltransferases, RND antibiotic efflux, gene regulatory resistance, ABC transporters, and nucleotidyltransferases. Among these, acetyltransferases and nucleotidyltransferases are members of the aminoglycoside class of antibiotics (Table 2).

## 4. Discussion

Soil bacterial communities in the Antarctic Peninsula region are dominated at the phylum level by Ascomycota, Actinobacteria, and Bacteroidetes. Significant differences exist in the distribution of genera across different regions (Figure 2). Specifically, *Pseudomonas*, *Salmonella*, *Nocardia*, and *Pseudochrobactrum* exhibited the lowest relative abundance in soil samples from the MS area, followed by the SI area. In contrast, the highest relative abundance occurred in soil samples from the DI area. Related studies have demonstrated that these five genera can degrade hydrocarbons and other pollutants [30,31,32,33]. In soil samples from four regions of the Antarctic Peninsula, particularly at the MS, *Bradyrhizobium* was the dominant species, with relative abundances exceeding 5%. At high latitudes, *Bradyrhizobium* can fix nitrogen, providing a nitrogen source for other heterotrophic microorganisms. The unique adaptations of these microorganisms to low temperatures, high salinity, and radiation may lead to new applications in various industries, underscoring the importance of studying microbial diversity in these regions [34,35,36]. *Mesorhizobium* may also dominate due to its nitrogen-fixing ability and environmental adaptability. The prevalence of *Mesorhizobium* in the soils of the MS could stem from its metabolic potential and adaptability, which far surpasses the other microbial genera and influences nitrogen cycling, nutrient cycling, and ecological processes in the station’s soils [37]. This study revealed variations in sample sizes across different locations. These discrepancies arose from geographic and weather conditions, as well as sampling strategies. King George Island exhibited a smaller sample size due to its complex geology and the presence of lava flows, which hindered sample collection. Similarly, weather conditions on Devil’s Island limited access to some planned sampling sites. However, we employed a systematic sampling strategy and conducted statistical analyses to ensure the reliability and comparability of our findings. Future research should refine the sampling strategy to mitigate the effects of sample size variations.

Proteobacteria, Actinobacteria, and Bacteroidetes frequently dominate soil bacterial communities in the four regions of the Antarctic Peninsula. At the genus level, most strains of the prevalent genera, apart from *Bradyrhizobium*, commonly degrade polycyclic aromatic hydrocarbons (PAHs) [38,39,40]. In the SI region, the relative abundance of bacterial genera such as *Nitrosomonas* and *Nitrospira* was high, making them the dominant ammonia-oxidizing bacteria (AOB) in the soils of the region. The research indicated a notable rise in the share of *Dyella* within the soil microbial community following the freeze-thaw cycle. The greater prevalence of *Dyella* in the SI region relative to the MS region highlights the necessity for additional investigation into freeze-thaw conditions within this area [41]. Multiple types of *Klebsiella* are potential opportunistic pathogens and may carry genes that lead to antibiotic resistance. However, *Klebsiella* are also capable of producing some key molecules that are beneficial to the organism, such as ammonia (NH_3_) and acetamide, and they also function in phosphorus solubilization and nitrogen fixation [42]. It was found that as the membrane lipid content of *A. aptamericus* increased at low temperatures, the membrane area increased, and the membrane lipids of *A. aptamericus* had a higher content of phosphate esters and neutral esters, which enabled the membrane lipids to maintain the liquid crystalline state at low temperatures [43]. A related study on the enzyme aptamer produced by aptameric bacteria found that at low temperatures, aptameric enzymes are more flexible than their mesophilic and hyperthermic counterparts because of the reduction in the number of salt bridges, arginine, and proline residues in their structures, which reduces the hydrophobicity of the enzyme and increases its hydrophilicity, leading to enhanced solvent–solvent interactions [44]. The rate of protein degradation in cryotolerant bacteria is fast compared to mesophilic bacteria. Rapid conversion of proteins is one of the ways of conserving energy under oligotrophic conditions in cryotolerant bacteria [45]. Thus, it is evident that membrane transporter functions as well as amino acid transport and metabolism functions help microorganisms to adapt to cold and oligotrophic conditions.

Based on KEGG and COG functional annotations, a high abundance of functions related to membrane transport, carbohydrate metabolism, amino acid transport, and metabolism, replication, recombination, and repair, and cell wall/membrane/envelope bioformation were found in the Antarctic Peninsula soil microbial community. The top twenty species in the correlation network diagram mainly belong to Proteobacteria, Actinobacteria, and Firmicutes (Figure 3). These species play key roles in various ecosystems. Proteobacteria were involved in the nitrogen cycle, sulfur cycle, and organic matter decomposition [46,47]. Actinobacteria break down complex organic matter in the soil and form symbiotic relationships with plant roots [48,49]. Firmicutes break down complex carbohydrates in animal intestines and are involved in energy metabolism [50]. Membrane transport functions, as well as amino acid transport and metabolism functions, are crucial for microorganisms to adapt to cold and oligotrophic environments. Furthermore, studies have shown that UV radiation impacts the abundance and diversity of microorganisms, such as bacteria and fungi, in soil, leading to DNA damage [51]. Replication, recombination, and repair functions play a significant role in the response of Antarctic soil microorganisms to elevated UV radiation intensity [52].

The Marambio Island study revealed a high level of COG annotation, particularly in secondary metabolite biosynthesis, translocation, and catabolism. This finding indicates a significant presence of genes related to secondary metabolites in the analyzed microbial genomes. The high level of COG annotation suggests that this microbial population possesses an efficient transporter system, such as ABC transporter proteins, which serve as a pivotal mechanism for antibiotic resistance [53,54]. Thus, this implies that microbial systems in this environment typically enhance their adaptations by translocating secondary metabolites. This process enables them to manage various environmental stresses by catabolizing multiple antibiotics or other secondary metabolites, consequently deriving additional nutrients from them. Human-induced activities significantly contribute to the emergence of antibiotic resistance in bacterial populations. However, bacterial communities produced antibiotics and resistance mechanisms long before human discovery, and these processes can manifest in regions devoid of human impact [55]. Furthermore, a related investigation revealed increased abundance and diversity of antibiotic resistance genes (ARGs) in freshwater samples collected from the heavily human-affected King George Island area of Antarctica [56].

Furthermore, microbial samples from the Antarctic Peninsula annotated with CAzy (Figure 9) demonstrated that glycoside hydrolases (GHs) [57] and glycosyltransferases (GTs) constituted a substantial portion of various microbial functions. Glycoside hydrolases are widespread in nature and play a crucial role in the degradation of cellulose specifically [58]. In microbial communities, glycoside hydrolases and glycosyltransferases work together to facilitate material cycling in ecosystems, degrading polysaccharides and resynthesizing them. Upon entering the cell, these sugar molecules participate in energy production and other biosynthetic activities, such as constructing cell walls and extracellular polysaccharides. This collaborative symbiotic relationship among different microorganisms enhances the metabolic efficiency of the entire community and its viability [59,60]. These characteristics illustrate how microorganisms have adapted to extreme environments like the Antarctic Peninsula by refining their own traits, while also enriching our understanding of organisms that thrive under extreme conditions, such as the phylum Proteobacteria and the phylum Actinobacteria.

## 5. Conclusions

This study found that in the more active geographic areas (e.g., MS), the species diversity and richness of the soil microbial communities were lower, while the dominance of the dominant species was more pronounced. Based on COG and KEGG functional analyses, the dominant microorganisms in the Antarctic Peninsula exhibit high levels of geomembrane transport, amino acid transport, and metabolic functions. These findings indicate that soil microorganisms in extreme environments adapt to exogenous factors by adjusting their molecular levels. The analysis of antibiotic resistance genes revealed that the main antibiotic resistance genes in Antarctic soil microorganisms were ABC Transporter (33.95%), Gene Modulating Resistance (23.02%), and RND Antibiotic Efflux Pump (23.02%). The relevant genes were aminoglycosides, β-lactamases, ribosomal RNA methyltransferases, antibiotic efflux, Gene Modulating Resistance, and ABC Transporter. Indirectly, the impact of antibiotics in the natural environment is subtle, even in regions where antibiotics are not widely used. Such insights highlight the intricate interplay between microbial adaptation, genetic diversity, and environmental factors. Focusing on the contributions of indigenous microorganisms that are adapted to extreme conditions is critical to understanding polar ecosystem functioning in the context of global climate change.

## Figures and Tables

**Table 1:** Sample information.

Sample Number	Sampling Area	Longitude	Latitude	Altitude (m.a.s.l)	Sampling Time	Accession No.
KGI-3-1	KGI	−57.7076	−62.0197	7	31 January 2020	SRR22742764
KGI-4	KGI	−57.7076	−62.0197	8	31 January 2020	SRR22742763
KGI-7-1	KGI	−57.7078	−62.0199	9	31 January 2020	SRR22742752
KGI-11	KGI	−57.7062	−62.0213	10	31 January 2020	SRR22742745
KGI-12	KGI	−57.7062	−62.0213	10	31 January 2020	SRR22742744
KGI-13	KGI	−57.7058	−62.0214	14	31 January 2020	SRR22742743
KGI-14	KGI	−57.7056	−62.0215	17	31 January 2020	SRR22742742
KGI-15	KGI	−57.7054	−62.0217	23	31 January 2020	SRR22742741
KGI-16	KGI	−57.7054	−62.0219	23	31 January 2020	SRR22742740
KGI-19	KGI	−57.7042	−62.0225	41	31 January 2020	SRR22742739
KGI-20	KGI	−57.6983	−62.0213	86	31 January 2020	SRR22742762
KGI-21	KGI	−57.6946	−62.0212	123	31 January 2020	SRR22742761
MS-31	MS	−56.6122	−64.2539	34	2 February 2020	SRR22742760
MS-34	MS	−56.6118	−64.2536	26	2 February 2020	SRR22742759
MS-37	MS	−56.6118	−64.2534	19	2 February 2020	SRR22742758
MS-40	MS	−56.6108	−64.2531	13	2 February 2020	SRR22742757
MS-41	MS	−56.6108	−64.2531	15	2 February 2020	SRR22742756
MS-42	MS	−56.6108	−64.2531	24	2 February 2020	SRR22742755
MS-43	MS	−56.6108	−64.2530	22	2 February 2020	SRR22742753
MS-45	MS	−56.6108	−64.2530	25	2 February 2020	SRR22742754
SI-50	SI	−56.68889	−64.2899	8	4 February 2020	SRR22742751
SI-51	SI	−56.68951	−64.2897	11	4 February 2020	SRR22742750
SI-52	SI	−56.6914	−64.2894	9	4 February 2020	SRR22742748
SI-53	SI	−56.6906	−64.2896	8	4 February 2020	SRR22742749
DI-68	DI	−57.2944	−63.7997	78	5 February 2020	SRR22742747
DI-71	DI	−57.2901	−63.8022	23	5 February 2020	SRR22742746

**Table 2:** Resistance genes.

Resistance Gene	Mechanism Classification
*AAC6-Ia*	Acetyltransferase
*Subclass B2*	β-lactamase
*VIM*	β-lactamase
*SubclassB1*	β-lactamase
*ErmA*	rRNA Methyltransferase
*adeA-adeI*	RND Antibiotic Efflux
*mecR1*	Gene modulating resistance
*ErmC*	rRNA methyltransferase
*TEM*	β-lactamase
*tolC*	ABC transporter
*AAC6-Ib*	Acetyltransferase
*ANT*	Nucleotidyltransferase

## Data Availability

SRA data generated in this study was uploaded in NCBI database. Project number PRJNA912251. BioSample accessions SAMN32236231, SAMN32236232, SAMN32236233, SAMN32236234, SAMN32236235, SAMN32236236, SAMN32236237, SAMN32236238, SAMN32236239, SAMN32236240, SAMN32236241, SAMN32236242, SAMN32236243, SAMN32236244, SAMN32236245, SAMN32236246, SAMN32236247, SAMN32236248, SAMN32236249, SAMN32236250, SAMN32236251, SAMN32236252, SAMN32236253, SAMN32236254, SAMN32236255, and SAMN32236256.
